# Operations for Cleft Palate

**Published:** 1907-09

**Authors:** 


### OPERATIONS FOR CLEFT PALATE.

Makuen summarizes his paper as follows: An operation for the closure of a cleft palate should be done only when there is a fair likelihood of success. It should be done only by those having acquired special skill in nasopharyngeal and oral surgery. When it is probable that several operations may be necessary the parents or patient should be so informed. The operation should be done as early as possible. In the difficult adolescent cases the operation after a preliminary tracheotomy, as described by Dr. McKennon, may be preferable. There are two reasons for attempting to close a clept palate, namely to, improve the general health of the patient and to increase the efficiency of the faculty of speech. The general health of the patient is benefited in two ways, namely, by improving the hygiene of the nasopharynx and the oral cavity, and by improving the general morale of the patient. The speech is improved by a course of pychophysical training, in which the patient is taught first to recognize normal speech and then to make the best use of his still imperfect organs in its production.—New York Medical Journal, July 27, 1907.

